# Metagenomic analysis of primary colorectal carcinomas and their metastases identifies potential microbial risk factors

**DOI:** 10.1002/1878-0261.13070

**Published:** 2021-08-30

**Authors:** Luigi Marongiu, Jonathan J. M. Landry, Tobias Rausch, Mohammed L. Abba, Susanne Delecluse, Henri‐Jacques Delecluse, Heike Allgayer

**Affiliations:** ^1^ Department of Experimental Surgery – Cancer Metastasis Medical Faculty Mannheim Ruprecht‐Karls University of Heidelberg Mannheim Germany; ^2^ Genomics Core Facility European Molecular Biology Laboratory (EMBL) Heidelberg Germany; ^3^ Division F100 German Cancer Research Center (DKFZ) Heidelberg Germany

**Keywords:** bacteriome, colorectal cancer, metastasis, phages, viruses

## Abstract

The paucity of microbiome studies at intestinal tissues has contributed to a yet limited understanding of potential viral and bacterial cofactors of colorectal cancer (CRC) carcinogenesis or progression. We analysed whole‐genome sequences of CRC primary tumours, their corresponding metastases and matched normal tissue for sequences of viral, phage and bacterial species. Bacteriome analysis showed *Fusobacterium nucleatum*, *Streptococcus sanguinis*, *F. Hwasookii*, *Anaerococcus mediterraneensis* and further species enriched in primary CRCs. The primary CRC of one patient was enriched for *F. alocis*, *S. anginosus, Parvimonas micra* and *Gemella* sp. 948. Enrichment of *Escherichia coli* strains IAI1, SE11, K‐12 and M8 was observed in metastases together with coliphages enterobacteria phage φ80 and Escherichia phage VT2φ_272. Virome analysis showed that phages were the most preponderant viral species (46%), the main families being *Myoviridae*, *Siphoviridae* and *Podoviridae*. Primary CRCs were enriched for bacteriophages, showing five phages (Enterobacteria, Bacillus, Proteus, Streptococcus phages) together with their pathogenic hosts in contrast to normal tissues. The most frequently detected, and Blast‐confirmed, viruses included human endogenous retrovirus K113, human herpesviruses 7 and 6B, Megavirus chilensis, cytomegalovirus (CMV) and Epstein–Barr virus (EBV), with one patient showing EBV enrichment in primary tumour and metastases. EBV was PCR‐validated in 80 pairs of CRC primary tumour and their corresponding normal tissues; in 21 of these pairs (26.3%), it was detectable in primary tumours only. The number of viral species was increased and bacterial species decreased in CRCs compared with normal tissues, and we could discriminate primary CRCs from metastases and normal tissues by applying the Hutcheson *t*‐test on the Shannon indices based on viral and bacterial species. Taken together, our results descriptively support hypotheses on microorganisms as potential (co)risk factors of CRC and extend putative suggestions on critical microbiome species in CRC metastasis.

## Graphical abstract

This work analysed the presence of sequences of bacteria and viruses in primary colorectal cancer (CRC) tissues, compared with corresponding normal colorectal tissues and metastases. We observed a higher viral species diversity and lower bacterial species diversity in primary CRCs than in matched normal tissues. Some microbial species were enriched in primary CRCs and some metastases, particularly phages.

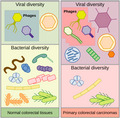

**Abbreviations**
AcMNPV: autographa californica nucleopolyhedrovirus
CD: Crohn’s disease
CMV: cytomegalovirus
CRC: colorectal cancer
EBV: Epstein–Barr virus
EMCV: encephalomyocarditis virus
HERV‐K113: human endogenous retrovirus K113
HHV‐6B: human herpesvirus 6B
HHV‐7: human herpesvirus 7
HPV: human papillomavirus
HPyV7: human polyomavirus 7
IBD: inflammatory bowel disease
JCV: JC virus
SV40: simian virus 40
UC: ulcerative colitis
VHD: Virus‐Host Database
WGS: whole‐genome sequencing

## 1 Introduction

Colorectal cancer (CRC) is the third most prevalent form of cancer worldwide, with a global incidence of over 1.4 million cases annually [1]. In Europe, it represents the second most frequent cause of death among people who have cancer and its incidence is increasing among young adults, who are not included yet in screening campaigns [2, 3]. About 3–5% of CRC cases show hereditary transmission [4, 5, 6], implying that the vast majority of patients develop this disease by accumulating molecular changes and further CRC‐promoting factors during their lifetime. Every person has a four per cent lifetime risk of developing sporadic CRC [7] but several studies have indicated the possibility of a microbial cofactor to increase such a risk [8, 9, 10]. In particular, it has been proposed that some bacterial species (microbial ‘drivers’), which carry genes encoding proteins that can induce chromosomal instability, might be able to initiate the oncogenic cascade in the intestinal cells [11, 12, 13]. Subsequently, other opportunistic bacteria (‘passengers’) could become more prevalent in this pretumour microenvironment, boosting inflammation and fostering oncogenesis or even progression. So far existing studies differ in drivers and passenger definitions, but the species *Fusobacterium nucleatum*, *Bacteroides fragilis*, *Streptococcus bovis* and *Enterococcus faecalis* are the most frequently cited [14].

Moreover, viruses could play a yet underestimated role in the carcinogenic process. In the past decades, several studies have pointed out an increased prevalence of viral infections in patients suffering from CRC or associated inflammatory conditions, such as inflammatory bowel disease (IBD), which include Crohn’s disease (CD) and ulcerative colitis (UC) [15]. IBD is a risk factor for CRC whose prevalence is currently increasing worldwide also [16, 17]. Among the viruses most frequently investigated for their possible association with CRC and IBD are Epstein–Barr virus (EBV, also known as human herpesvirus type 4, HHV‐4), cytomegalovirus (also known as human herpesvirus type 5, HHV‐5), human papillomavirus (HPV) and several members of the *Polyomaviridae* family [18, 19, 20, 21]. Nevertheless, a causative involvement of viruses in CRC oncogenesis or progression is still unclear [21].

Recent microbiome work has expanded beyond human‐infecting viruses and has highlighted, for example, the importance of bacteria‐infecting viruses (phages) in CRC and IBD. For instance, an increased prevalence of phage species belonging to the order of *Caudovirales* (which includes the families *Siphoviridae*, *Myoviridae* and *Podoviridae*) has been observed in patients affected by these diseases [22, 23, 24, 25]. Phages can indirectly contribute to CRC development by modulating the prevalence of driver and passenger bacteria [26, 27, 28, 29, 30, 31, 32]. Diet is also crucial in this context because it can alter the bacterial composition and, consequently, that of the phages [33]. This association can, at least in part, explain the epidemiological link observed between a western type of nutrition, which is usually poor in fibres but rich in fats and red meat, and a higher risk of CRC [10]. Furthermore, most microbiome studies have been based upon faecal samples, which can be considered a proxy to the actual microbial community present within the intestinal tissue. Since mucus covers the intestine walls, acting as a barrier to the microorganisms [34, 35], faecal and tissue specimens do not necessarily bear the same microbial species [36]. The few microbiome studies based on tumour tissues have shown a higher prevalence of phages in inflammatory lesions than in normal matched sections [37, 38].

The paucity of microbiome studies based on intestine tissues has contributed to the poor understanding of the conditions that foster CRC. Specifically, there is certainly a lack of research based on metastases. About one‐quarter of CRC cases progress towards metastasis [39, 40]; thus, understanding whether microorganisms modulate this progression could help to determine cofactors and consequently improve the diagnosis and treatment of metastases.

In this study, we (re‐)analysed whole‐genome sequences (WGS), which we generally described in previous work [41], to assess whether we could detect the enrichment of particular microbial sequences in primary colorectal carcinomas in comparison to matched control tissues, and whether metastases show additional microbial sequences that could potentially set them aside from colorectal primary tumours. This metagenomic analysis might help not only to further understand, or suggest, putative microbial cofactors in the oncogenic and metastatic process, but support in the diagnosis or even risk prediction for CRC carcinogenesis or metastasis.

## 2 Materials and methods

### 2.1 Tissues

All of the samples were completely anonymized and handled exclusively in strictly anonymized conditions. Tissues had been generally obtained from the biobank of the Medical Faculty Mannheim (Dept. of Surgery), University of Heidelberg, Germany, and the biobanking and tissue sampling approved by the Ethical Committee of this institution. The primary colorectal tumours, matched healthy colorectal tissues, and corresponding metastases re‐analysed here were described previously [41], the analysis having been undertaken with the understanding and written consent of each subject (or relatives if deceased), and the Declaration of Helsinki was followed. Samples were processed and anonymized data stored within the EGA database (accession number EGAS00001002717) as already described [41]. Genomic DNA had been isolated from frozen tissue sections with the QIAamp DNA mini kit (Qiagen, Hilden, Germany), according to the manufacturer’s instructions. The extracted DNA had been quality controlled and sequenced as described previously [41] at the Genomics and Proteomics Core Facility of the German Cancer Research Center (DKFZ), Heidelberg, Germany, on the Illumina HiSeq2000 platform.

### 2.2 Virome analysis

Genomic sequences from the human assembly issue 38 built 12 (GChr38) were obtained from the *Ensembl* database. Viral reference sequences (*n* = 10 384) were retrieved from the National Center for Biotechnology Information (NCBI) database using *Entrez* utilities (https://www.ncbi.nlm.nih.gov/books/NBK25497/) and were concatenated into a single sequence (viral chromosome) using mingle v. 2.3 [42]. Human and viral chromosomes were combined into a single fusion reference genome indexed with bwa v. 0.7.17 [43].

Sequencing adapters and reads with quality below 33 phred‐score units were removed with trimmomatic v. 0.38 [44, 45, 46]. The remaining reads were aligned against the composite human/virus reference genome with bwa‐mem v. 0.7.17 [47]. De‐duplication was carried out with sambamba v. 0.6.7 [48], and only reads mapping to the virus chromosome, and with mapping quality of at least ten phred‐score units, were retained. The starting point of the remaining reads was used in combination with the index file provided by *Mingle* to determine the corresponding viral species. Reads mapping to phage φX174, which was used as quality control in the libraries’ preparation, were discarded.

The quality of the alignment and sample coverage was assessed with samtools v. 1.9‐18 and qualimap v. 2.2.1 [49, 50]. blast+ v. 2.9.0 [51, 52] was used to align the remaining reads against the human chromosome, the composite viral chromosome alone, or the individual virus genomes. Reads that aligned preferably to the human genome were removed. For each sample, overlapping reads belonging to the same viral species were merged into a single consensus sequence using clustal omega v. 1.2.4 [53]. To further remove possible false‐positive results, these consensuses were aligned against the NCBI protein database with blastx v. 2.9.0 [54]. Sequences identified as nonviral were discarded. For each specimen, remaining consensus sequences belonging to the same virus were concatenated into a single viral species. Structural variation (SV) was investigated with delly v. 0.8.3 [55].

In a series of 80 colorectal primary tumours and corresponding normal colorectal tissues, EBV DNA was detected as a validation by end‐point PCR targeting EBNA1 as previously described [56]. Briefly, total cellular RNA was isolated with RNeasy Mini Kit (Qiagen) according to the manufacturer’s instruction and the cDNA generated using SuperScript III (Thermo Fisher Scientific, Karlsruhe and Dreieich, Germany) as recommended by the manufacturer. Amplification of the cDNA was accomplished with the Taq polymerase kit provided by Thermo Fisher Scientific (Cat. No. EP0401) with the following conditions: 2.5 μL of reaction buffer 10×, 2 mm of each dNTP, 1 μm of forward primer (5´‐CCGCTCCTACCTGCAATATCA‐3´), 1 μm of reverse primer (5´‐CAATAACGGCAGCAAGCTTG −3´), 1 mm MgCl_2_, 1.25 units of enzyme, 100 ng template DNA and water to 25 μL. The amplification conditions were as follows: 5 min at 95 °C followed by 30 cycles of 1 min at 95 °C, 1 min at 58 °C, 1 min at 72 °C and final extension for 7 min at 72 °C.

To address the question of cell types primarily affected within these tissues, the presence of EBV was detected by in situ hybridization with an EBER‐specific peptide nucleic acid probe, in conjunction with a PNA detection kit (Dako) following the manufacturer’s protocol as described [57]. Images were taken with a camera attached to a light microscope (M2500; Leica).

### 2.3 Bacteriome analysis

Reads not mapping to either the human chromosomes or the composite virus chromosomes were assembled with spades v. 3.13.0 [58]. Sequence assemblies smaller than 1000 base pairs were discarded; assembled sequences identified as metazoan, artificial or environmental sequences were removed. The classification of the obtained sequences was kept at the species level and subspecies and strains were merged together. The bacteria‐phage infection network was assessed by inquiring the *Virus‐Host Database* (VHD) [59]. Classification of phages as lytic or virulent was based on the *PhageAI* database [60].

### 2.4 Statistical analysis

Data processing was performed with julia v. 1.3.1 (https://julialang.org/) and bash v. 5.0.3 languages. Plotting, statistical analysis and hierarchical clustering were performed with r v. 3.6.1 [61]. The Shannon index (or, more appropriately, Shannon‐Wiener index or entropy) [62] and its variance were computed with the r package *Qsutils* [63]. Species richness analysis [64, 65] was obtained with the r package *iNEXT* [66]. Statistical comparison between sample groups was carried out by paired *t*‐test. The Hutcheson *t*‐test [67] was used to compare Shannon indices, and it was implemented in r with the package ecoltest (https://github.com/hugosal/ecolTest).

## 3 Results

### 3.1 Virome analysis

**Fig. 1:**
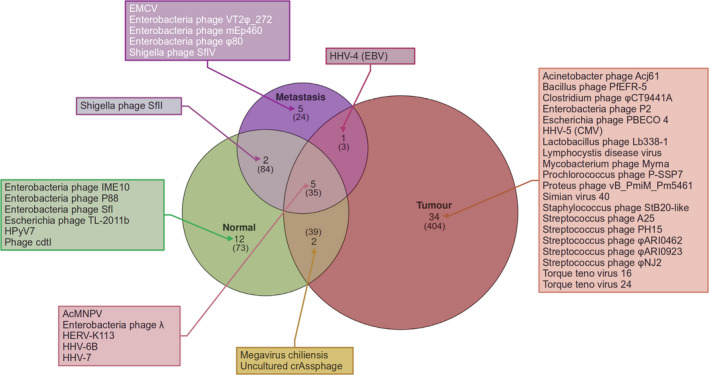
Stratification of viral species sequences observed in the present sample set. Venn diagram showing the number of virus species present within each tissue type (normal tissue, primary tumour and metastasis) based on the Blast‐filtered data. The number of species observed prior to this filtering step is given in parentheses. For each group, the most representative species are reported.

Twelve cases previously described [41] with matched primary colorectal tumours, corresponding liver (or lung in one case) metastasis and normal colorectal tissues were available for the present analysis. After sequencing, the subset of reads mapping to viral genomes had a mean coverage of 28.33 ± 16.21 (Table S1). We initially identified 662 viral species of which 272 (41.1%) were phagial with the families *Myoviridae* (*n* = 154), *Siphoviridae* (*n* = 79) and *Podoviridae* (*n* = 29) as the most represented (Fig. 1, Table 1). The most prevalent species were human endogenous retrovirus K113 (HERV‐K113), Synechococcus phage S‐SM2, Enterobacteria phage λ and autographa californica nucleopolyhedrovirus (AcMNPV). Among the most represented DNA viruses, human herpesvirus 7 (HHV‐7) was observed in eight, and HHV‐6B in six specimens, besides Ebstein‐Barr virus (EBV, HHV‐4) and Cytomegalovirus (CMV or HHV‐5). Several genotypes of torque teno (TT) virus were identified: 5, 9, 16, 24, 7, 11 and 5. One sample showed sequences of simian virus 40 (SV40) and another bore human polyomavirus 7 (HPyV7). We also identified several giant viruses such as Pandoravirus salinus and dulcis, Megavirus chiliensis, Cafeteria roenbergensis and Acanthamoeba polyphaga. Among the phages, we observed the uncultured crAssphage in six tissues. There were also several RNA species (*n* = 189), including the aforementioned HERV‐K113 (an endogenous retrovirus) and encephalomyocarditis virus (EMCV). There were 73, 404 and 24 viral species present either in normal tissues, primary colorectal tumours, or metastases only, respectively, and an additional three (27.3%) were common to both primary colorectal tumours and metastases: EBV, Qinghai Himalayan marmot astrovirus and Tipula oleracea nudivirus. EBV was present in the lung metastasis together with AcMNPV, Synechococcus phage S‐SM2 and HERV‐K113.

**Table 1:** Selection of the most represented viral species based on raw data (reads aligned only by BWA‐MEM) and assignment to their respective families.

Group	Virus	Family	Type
Normal, tumour and metastasis	HERV‐K113	*Retroviridae*	Endogenous retrovirus
	AcMNPV	*Baculoviridae*	DNA virus
	Enterobacteria phage λ	*Siphoviridae*	Bacteriophage
	Synechococcus phage S‐SM2	*Myoviridae*	Bacteriophage
	Escherichia phage TL‐2011b	*Podoviridae*	Bacteriophage
	Pandoravirus neocaledonia	*Pandoraviridae*	Giant virus
	HHV‐7	*Herpesviridae*	DNA virus
	Cafeteria roenbergensis virus	*Mimiviridae*	Giant virus
	Pandoravirus salinus	*Pandoraviridae*	Giant virus
	Phage cdtI DNA	*Siphoviridae*	Bacteriophage
	HHV‐6B	*Herpesviridae*	DNA virus
	Pandoravirus dulcis	*Pandoraviridae*	Giant virus
Normal and metastasis	Torque teno midi virus 5	*Anelloviridae*	DNA virus
	Torque teno midi virus 9	*Anelloviridae*	DNA virus
	Encephalomyocarditis virus	*Picornaviridae*	RNA virus
	Hepatitis C virus genotype 1	*Flaviviridae*	RNA virus
	Enterobacteria phage VT2φ_272	*Podoviridae*	Bacteriophage
	Shigella phage SfII	*Myoviridae*	Bacteriophage
	Escherichia phage pro483	*Myoviridae*	Bacteriophage
	Shigella phage SfIV	*Myoviridae*	Bacteriophage
	Enterobacteria phage mEp460	*Siphoviridae*	Bacteriophage
Normal and tumour	Megavirus chiliensis	*Mimiviridae*	Giant virus
	Uncultured crAssphage	*Unassigned*	Bacteriophage
	Aeromonas phage PX29	*Myoviridae*	Bacteriophage
	Enterobacteria phage P88	*Myoviridae*	Bacteriophage
	Enterobacteria phage P2	*Myoviridae*	Bacteriophage
	Acanthamoeba polyphaga mouvirus	*Mimiviridae*	Giant virus
Tumour only	CMV	*Herpesviridae*	DNA virus
	Streptococcus phage φARI0462	*Adenoviridae*	Bacteriophage
	Bacillus phage PfEFR‐5	*Siphoviridae*	Bacteriophage
	Proteus phage vB_PmiM_Pm5461	Myoviridae	Bacteriophage
	Streptococcus phage φARI0923	Siphoviridae	Bacteriophage
	Simian virus 40	*Polyomaviridae*	DNA virus
	Acinetobacter phage Acj61	*Myoviridae*	Bacteriophage
	Clostridium phage phiCT9441A	*Myoviridae*	Bacteriophage
	Escherichia phage PBECO 4	*Myoviridae*	Bacteriophage
	Lactobacillus phage Lb338‐1	*Herelleviridae*	Bacteriophage
	Lymphocystis disease virus	*Iridoviridae*	DNA virus
	Mycobacterium phage Myrna	*Myoviridae*	Bacteriophage
	Prochlorococcus phage P‐SSP7	*Autographiviridae*	Bacteriophage
	Staphylococcus phage StB20‐like	*Siphoviridae*	Bacteriophage
	Streptococcus phage A25	*Siphoviridae*	Bacteriophage
	Streptococcus phage PH15	*Siphoviridae*	Bacteriophage
	Streptococcus phage phiNJ2	*Siphoviridae*	Bacteriophage
	Torque teno virus 16	*Anelloviridae*	DNA virus
	Torque teno virus 24	*Anelloviridae*	DNA virus
Metastasis only	Enterobacteria phage HK629	*Siphoviridae*	Bacteriophage
	Enterobacteria phage HK97	*Siphoviridae*	Bacteriophage
	Enterobacteria phage M13	*Inoviridae*	Bacteriophage
	Enterobacteria phage P1	*Myoviridae*	Bacteriophage
	Enterobacteria phage φ80	*Siphoviridae*	Bacteriophage
Tumour and metastasis	EBV	*Herpesviridae*	DNA virus
	Tipula oleracea nudivirus	*Nudiviridae*	DNA virus
	Qinghai Himalayan marmot astrovirus	*Astroviridae*	RNA virus
Normal only	Enterobacteria phage IME10	*Podoviridae*	Bacteriophage
	Enterobacteria phage SfI	*Myoviridae*	Bacteriophage
	Human polyomavirus 7	*Polyomaviridae*	DNA virus

To avoid the possibility of false‐positive detection, we further filtered the data with the highly sensitive Blast alignment (see Materials and methods, [51, 52]). After Blast filtering, we found 61 viral species across all of the tissue entities that passed the threshold, corresponding to 9.2% of the initial species identified, of which 28 (45.9%) were phages, with the families *Siphoviridae* (*n* = 11), *Myoviridae* (*n* = 10) and *Podoviridae* (*n* = 7) as the most represented (Fig. 1). Of the 28 phagial species, 10 (35.7%) had *E. coli* as a host (coliphages) and 15 (53.6%) were observed in primary colon tumours. Stratification of the phagial species by tissue type showed that one species (Enterobacteria phage λ) was common to all tissues types, one (Shigella phage SfII) was common to normal colorectal tissues and liver metastases. Enterobacteria phage VT2φ_272 and enterobacteria phage φ80, Shigella phage SfIV and enterobacteria phage mEp460 were confirmed specifically in one metastasis each. An additional 16 species were present only in primary carcinomas, and six were observed only in normal colorectal tissues. Overall, 87.5%, 70.6% and 100.0% of phages in in normal tissues, primary colorectal tumours and liver metastases were temperate, respectively. Filtering confirmed HERV‐K113, AcMNPV, phage λ (but not Synechococcus phage S‐SM2) as the most preponderant viral species across all tissue entities. HHV‐7 was present collectively in five patients, and specifically in the normal colorectal tissue and primary colorectal carcinomas of two patients as well as in the primary colorectal carcinoma and liver metastasis of another patient. HHV‐6B was confirmed collectively in four patients, and specifically in the normal colon tissue and primary colorectal carcinoma of one patient as well as the normal colon tissue and liver metastasis of another patient. CMV was present specifically in the primary colorectal carcinoma tissues of two patients. Torque teno (TT) viruses 16 and 24 were also confirmed in primary colorectal carcinomas. We also confirmed SV40 and HPyV7 in one case each (in a primary colorectal tumour and one normal colon tissue, respectively). EMCV was specifically present in liver metastasis tissue. Of the aforementioned giant viruses, only megavirus chiliensis was confirmed in two patients. The phage crAssphage was confirmed in the normal colon tissues and the primary colorectal tumours of three patients. EBV was confirmed after BLAST filtering in the primary colorectal tumour and the lung metastasis of the same patient. Himalayan marmot astrovirus and Tipula oleracea nudivirus were not confirmed in the lung metastasis after Blast filtering.

**Fig. 2:**
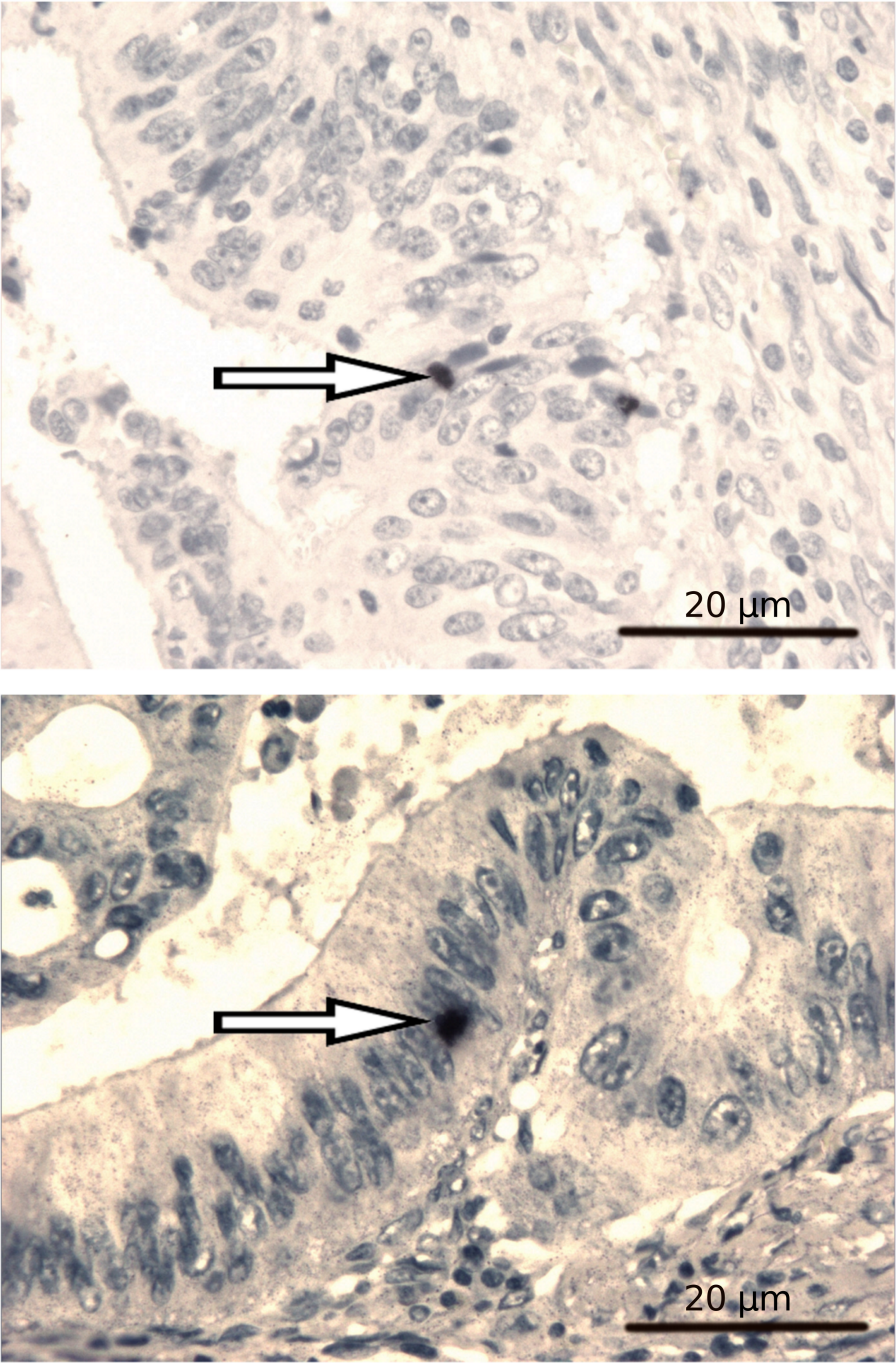
Visualization of EBV in primary colorectal cancer tissues with EBER staining. The pictures show an *in situ* hybridization with an Epstein–Barr expression region (EBER)‐specific probe performed on histological sections. EBV‐infected cells are depicted in black (examples shown with arrows). Magnification 400×, scale bar 20 μm.

Given the recognized oncogenic potential of EBV, we further investigated, and sought to validate, the frequency of presence of EBV by PCR on a group of 80 independent primary colorectal tumour and matched corresponding normal colorectal tissues (Table 2). Of 41 tumour samples positive in PCR, in 21 cases (51.2%) EBV was detectable specifically in the primary colorectal carcinomas but not in corresponding normal tissues, with a significant paired *t*‐test (*P* = 0.048). To gain additional information on putative cell types infected with EBV within colorectal carcinoma tissues, the presence of EBV was further investigated in a subset of 5 samples using EBER staining (ISH) (representative examples shown in Fig. 2). EBER staining of individually positive EBV‐cases (in PCR) showed positivity for the ISH probe in isolated cells with a small nucleus, which were tightly associated with neoplastic CRC glands. These images suggest infection of cancer‐associated lymphocytes, without definite evidence for epithelial cell infection, although, in a few instances, staining appeared projected onto single epithelial cells.

**Table 2:** Prevalence of EBV by PCR, *n* = 80 validation cases.

Tissue entity	Presence of EBV	
	Yes	No
Primary tumour	41	39
Normal tissue	30	50

**Fig. 3:**
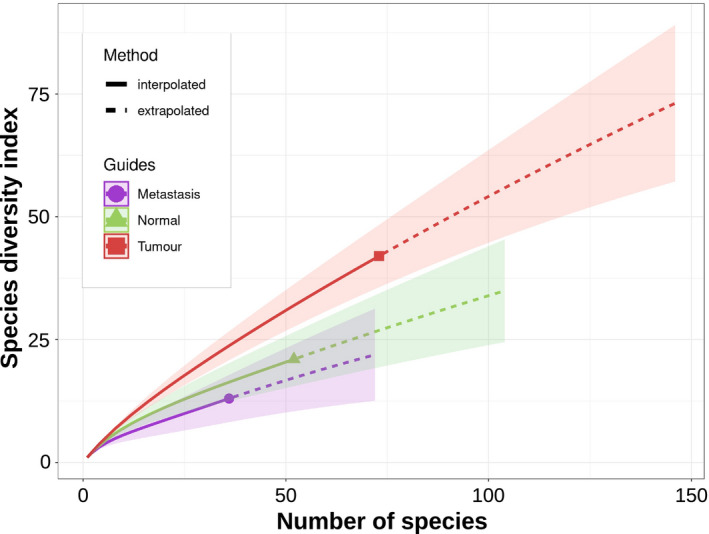
Viral richness. Rarefaction analysis of the viral species normalized for the sample size. Rarefaction is a bootstrap method that allows the direct comparison of samples by giving a count of species normalized for the sample size. The curves represent the mean measures of the number of species identified during the sampling process; the shaded areas depict the 95 confidence intervals of the measurements. Subspecies and strains were merged within the same species.

Next, we sought to determine whether we could differentiate primary colorectal tumour tissues, corresponding normal colorectal tissues, and metastases based on the viral content. We used rarefaction analysis to calculate the estimated number of species normalized for the sample size, and the Shannon index to have a comparative value. Overall, there were an estimated 124 (95% CI: 60–296), 232 (95% CI: 99–674) and 57 (95% CI: 26–157) normalized viral species, in the normal, primary colorectal tumour and metastatic tissues, respectively (Fig. 3). The respective species richness corresponded to Shannon indices of 2.56, 3.31 and 1.99, respectively. The Hutcheson t‐test allowed to discriminate the normal colorectal tissues from the primary colorectal tumours (*P*‐value < 0.001) and either liver or lung metastases (*P*‐value = 0.006) as well as primary colorectal tumours from either liver or lung metastases (*P*‐value < 0.001).

### 3.2 Bacteriome analysis

We identified 518 bacterial species, corresponding to 143 bacterial families in our sample set. Overall, there were 391, 217 and 10 bacterial species in normal colorectal tissues, primary colorectal tumours and liver metastases, respectively, corresponding to a ratio of phages over bacteria of 0.02, 0.08 and 0.60. There were 122 species present only in primary colon tumour tissues, four strains of *Escherichia coli* specifically in liver metastasis (IAI1, K‐12, M8 and SE11) and *Klebsiella pneumoniae* concomitantly in primary colon tumour and liver metastasis. There were no bacterial species observed in the lung metastatic tissue. We found *Fusobacterium nucleatum* enriched only in the primary colorectal tumour sections of three patients in contrast to corresponding normal colorectal tissues, whereas *Fusobacterium hwasookii* was present in the primary colorectal tumour sections of two patients. *Porphyromonas gingivalis* was present in the normal colorectal tissue and primary colon carcinoma sections of one patient. *Anaerococcus mediterraneensis* and *Prevotella denticola* were observed in the primary colorectal tumours of two patients, as opposed to corresponding normal tissues. Among the 122 species observed only in primary colorectal tumours, the families *Bacillaceae* and *Clostridiaceae* represented the most represented, with 20 species each (16.4%), followed by the *Streptococcaceae* with 17 species (13.9%). *Bacteroides fragilis* was prevalent in normal colorectal tissues and also present in primary colorectal tumour sections. *Streptococcus anginosus* was observed in the normal colorectal tissue of one patient, and in the primary colorectal tumours as opposed to their matched normal tissues of two patients. Furthermore, among the species observed only in primary colorectal tumours, *S. sanguinis*, *Filifactor alocis*, *Gemella* sp. oral taxon 928 and *Parvimonas micra* were present in high frequency in one patient and were represented by a number of sequence assemblies in the range of 468–147, compared to a mean of 5.7, indicating their highly specific representation.

**Fig. 4:**
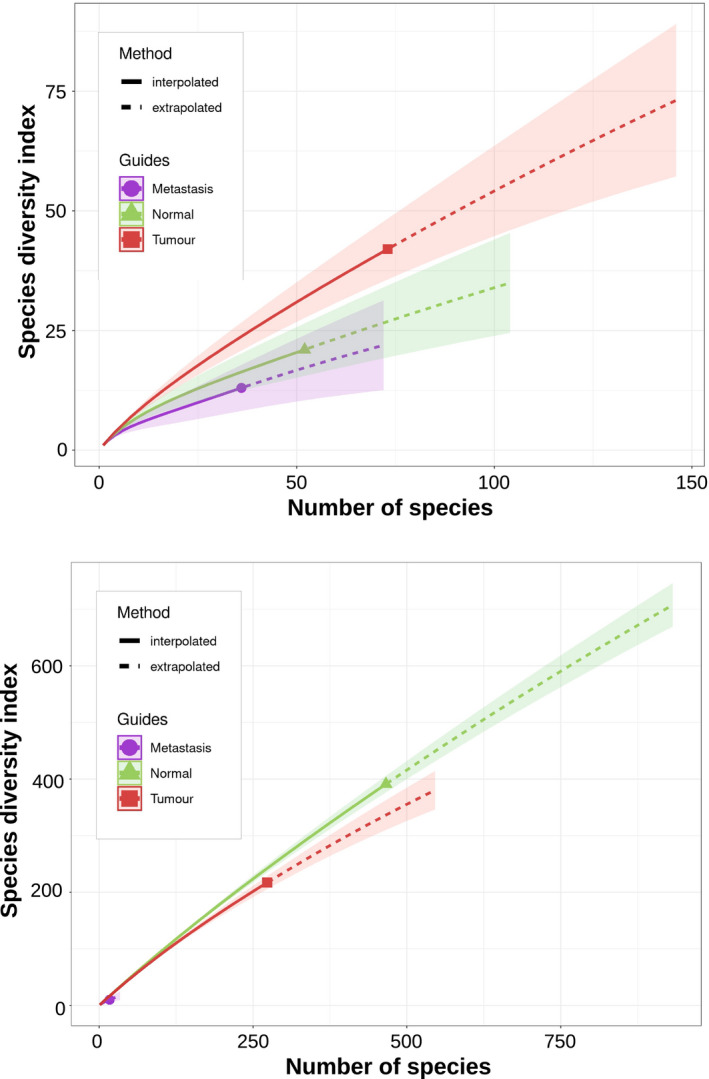
Bacterial richness. Rarefaction analysis of the bacterial species normalized for the sample size. Rarefaction is a bootstrap method that allows the direct comparison of samples by giving a count of species normalized for the sample size. The number of bacterial species in the metastases is much less than in normal colorectal and colorectal carcinoma tissues, reducing the curve to close to the coordinates 0, 0. The curves represent the mean measures of the number of species identified during the sampling process; the shaded areas depict the 95 confidence intervals of the measurements. Subspecies and strains were merged within the same species.

The normalized species count, obtained by rarefaction analysis, was 2370 (95% CI: 1701–3383), 1011 (95% CI: 693–1543) and 40 (95% CI: 14–209) for the normal, primary colorectal carcinoma and metastatic tissues (Fig. 4), corresponding to Shannon indices of 5.87, 5.27 and 1.95, respectively. The Hutcheson *t*‐test allowed discriminating normal tissues from both primary colorectal tumours (*P*‐value < 0.001) and metastases (*P*‐value < 0.001); similarly, primary colorectal tumours could be discriminated from metastases (*P*‐value < 0.001).

We further investigated the relationship between phages and bacteria by enumerating them in the tissues. The phagial hosts of the observed phages were derived from the Virus‐Host Database (VHD). Our results indicated a certain level of parallelism between the presence of the host and that of the associated phage (Table 3). Half of the eight phages observed most frequently had *E. coli* as a host. Enterobacteria phage λ was observed in all tissue types with a peak in the primary colorectal tumour sections. Enterobacteria phage P2 and φ80 were observed concomitantly with their host *E. coli* in one primary colorectal carcinoma and one liver metastasis, respectively. The primary tumour tissues also contained Bacillus phage PfEFR‐5 together with its host *Bacillus cereus,* as well as Streptococcus phage φARI0462 and φARI0923 together with their host *Streptococcus pneumoniae*.

**Table 3:** Parallelism between phages and their hosts. The simultaneous presence of sequences belonging to phages and their host within the same patient is reported.

Phage		Host	
Enterobacteria phage λ	(N, T, M)	*Escherichia coli*	(N, T, M)
Enterobacteria phage P88	(N)	*Escherichia coli*	(N)
Enterobacteria phage P2	(T)	*Escherichia coli*	(T)
Enterobacteria phage φ80	(M)	*Escherichia coli*	(M)
Bacillus phage PfEFR‐5	(T)	*Bacillus cereus*	(T)
Proteus phage vB_PmiM_Pm5461	(T)	*Proteus mirabilis*	(T)
Streptococcus phage phiARI0462	(T)	*Streptococcus pneumoniae*	(T)
Streptococcus phage phiARI0923	(T)	*Streptococcus pneumoniae*	(T)

M, liver metastasis; N, normal colon tissue; T, primary colon tumour.

## 4 Discussion

The present work is one of the very few, if not the first so far, that carried out microbiome analysis on primary colorectal carcinoma and corresponding metastases in comparison to matched normal tissues. Although, certainly, our analysis from whole genomes so far is largely bioinformatic, our study suggests that some microorganisms, at least their sequences, are more prevalent in some primary colorectal tumours or metastases, this pertaining to viruses and bacteria, and—potentially most interesting since these were not yet systematically investigated in such a setting—(bacterio‐)phages.

In both primary colorectal cancers and metastases, we observed several species of phages, for example phage λ or crAssphage, crAssphage having been reported as the most common virus in the colon [68, 69, 70]. The high prevalence of phages in general and, specifically, their enrichment in primary colorectal tumours matches previous studies indicating an increased prevalence of phages in CRC and IBD [22, 23, 24, 25, 37, 38]. The majority (over 45%) of the phages we observed had *E. coli* as a host, for example enterobacteria phage φ80 and Escherichia phage VT2φ 272 which, after Blast filtering, we even found specifically in metastatic tissues. We observed over half of the most frequent phages especially in primary tumours, two of which (Bacillus prophage PfEFR‐5 and Streptococcus phage φARI0462) showing their specific host (*Bacillus cereus* and *Streptococcus pneumoniae*, respectively) in primary tumour tissues only, not in corresponding normal tissues, which suggests that this might not have been due to contamination. This is hypothesized since the primary colorectal cancers and corresponding normal colorectal tissues were both acquired simultaneously, being exposed to the same microbiologic milieu of the same intestinal lumen within the same patient under the same settings, and therefore, they would have been exposed to the same contaminants. Interestingly, some of the hosts of the phages specifically observed in primary colorectal tumour tissues are known pathogens. For instance, Bacillus prophage PfEFR‐5 infects *Bacillus cereus*, a pathogen associated with food poisoning [71]. The observation of *B. cereus* and *S. pneumoniae* in tumour samples only, in contrast to the corresponding normal colorectal tissue of the very same patient, suggested a sort of ‘linkage disequilibrium’ between these tissue types.

The bacterial species we observed enriched, or exclusively, in primary colorectal carcinomas have been reported to show oncogenic or pro‐inflammatory potential. Our tumour‐specific findings of *F. nucleatum*, *S. anginosus* and *F. alocis*, *S. sanguinis*, *P. micra* and *Gemella* oral taxon 928 confirm the results of other research groups [72, 73, 74, 75, 76, 77, 78, 79, 80]. In particular, a recent microbiome analysis of CRC tissues found the species *F. nucleatum*, *S. anginosus* and *S. sanguinis* at high prevalence in cancer tissues (false discovery rate lower than 10^5^) [81]. *Fusobacterium* spp. are included in the passenger/driver model of CRC and have been reported to promote the formation of metastases [11, 82]. *F. nucleatum* and *P. micra* are commonly enriched in CRC sections [83, 84, 85], and a recent microbiome study reported the enrichment of *Fusobacterium hwasookii* and *Porphyromonas gingivalis* in colorectal cancer tissues, together with high prevalence of Fusobacteria and Bacteroidetes [86]. *F. alocis* and *P. micra* are prevalent at sites of periodontitis (a chronic inflammation of the gum that results in tooth loss) and in oral squamous cell carcinomas [79, 87, 88]. *S. sanguinis* has been reported in cases of occult colon carcinomas [89, 90, 91]. *Prevotella denticola* was enriched in colorectal cancer tissues [92]. *Klebsiella pneumoniae*, although commonly associated with pneumonia, has been reported as a risk factor for colorectal cancer [93, 94]. *S. anginosus* is an opportunistic bacterium commonly observed in the oral and intestinal flora, but it is also enriched in esophageal and gastric cancers [95, 96], and has been reported in a case of rectal adenocarcinoma [97]. *Gemella* is a genus of commensal bacteria of the mouth and gut whose main members (*G. morbillorum* and *G. haemolysans*) are usually isolated in abscesses and endocarditis [98, 99, 100]. *Anaerococcus mediterraneensis* has been recently isolated in a case of vaginosis [101], suggesting a potential pathogenic role or an association with dysbiosis.

Although *E. coli* was commonly observed in our samples, we also observed an enrichment of some *E. coli* strains specifically in metastatic tissue. *E. coli* is both a commensal of the human gut but also an opportunistic species [102]. Thus, lytic phages might remove commensal strains of *E. coli*, unbalancing the gut microbiome. On the other hand, phages can piggyback pathogenic species invading inner tissues. Therefore, our detection of coliphages might reflect these scenarios, and further data are needed to discriminate between these possibilities. Interestingly, it has been shown that this bacterium can use α‐haemolysin to invade the colon epithelium in a process known as ‘focal leak’ [103]. Specific strains of *E. coli* (C25 and HBTEC‐1) have been reported to transcytose colonic cells [104]. The portal vein directly connects the human gut to the liver, establishing a special connection between these two organs [105]. It has been shown that dysbiosis and inflammation enhance bacterial translocation, that is, the movement of bacteria and their products from the intestinal lumen to the mesenteric lymph nodes, the bloodstream and, consequently, the liver [106]. The intestinal barrier is also negatively affected by excessive food intake [107, 108], which also can trigger dysbiosis [109]. In turn, increased bacterial translocation can affect the liver, for instance by causing cirrhosis [110]. An impaired intestinal barrier can facilitate, alongside bacteria, the translocation of phages as well, an event that is believed to occur naturally [111].

The gut and the liver share a special relationship due to their connection via the portal vein, the biliary tract and systemic circulation. Hence, the presence of pathogenic bacteria in the gut might be reflected in the liver especially if there are leakages in the mucosal barrier in the intestine. Such a hypothesis is backed by extensive literature, demonstrating a linkage between intestinal dysbiosis and liver disease due to an increased permeability of the intestinal barrier. If certain bacteria or phages (viruses), or their disbalance, can be pathogenic for the colorectal tissue, they might affect the liver, too, in a similar or also slightly different way as compared to the intestinal tissue. Specifically, if particular pathogens, or their interaction, are able to induce inflammation in the gut, they might also induce inflammatory processes in the liver. However, with the exception of some specific systemically infecting viruses such as HCV, EBV, and others which are able to induce pro‐oncogenic pathways in cells including hepatocytes [112, 113, 114], still very little data are available showing how microbiobal pathogens translocated into the liver might foster cancer or cancer metastasis.

Nevertheless, the implications for the presence of bacteria and phages in the liver are still poorly understood. It is feasible to assume that these microorganisms might induce a chronic inflammation that can foster liver disease, albeit more experimental data are required to determine whether microorganisms might also induce metastasis. The fact that the ratio of phages to bacteria was much higher in metastases than in colorectal tissues might reflect the fact that phages are more likely to cross the gut epithelial barrier. Recent metagenomic analysis suggested that the induction of temperate phages might be responsible for propagating intestinal dysbiosis, resulting in an increased amount of phages in relation to bacteria [27, 115, 116]. The higher phage‐to‐bacteria ratio observed in primary colorectal tumours and liver metastases, including 100% temperate phages in the latter, might be explained in this context. Further experiments might help determine the phage‐to‐bacteria ratio with greater accuracy than the present work.

Furthermore, recent metagenomic studies have reported the increased prevalence of members of the *Enterobacteriaceae* family (thus, including *E. coli*) in the inflamed gut [117]. Our observations, therefore, can be explained in such a context, but more experimental work is needed to prove that such presence is not coincidental or to analyse whether the transmission of suchlike species, including possibly their phages, through leaky gut situations into other organs is contributing to the ‘seed’ or ‘soil’ component within any metastatic process. Along these lines, strains of *E. coli*, in addition to *B. fragilis*, *E. faecalis* and *F. nucleatum*, all of which we found represented in our tissues analysed, have been suggested to be able to act as pathogens, questioning their in part uncritical use as probiotics [78, 118, 119, 120, 121]. Several members of the *Enterobacteriaceae* family, including strains of *E. coli* and *K. pneumoniae*, have been shown to release a genotoxin (colibactin) able to induce genetic damage, fostering the insurgence of cancer [122, 123].

The oncogenic potential of these bacteria, together with, at least in part, an enrichment in primary colorectal carcinomas we observed herein, suggest a putatively complex scenario of several infectious components which, in their interaction, might lead to a netto support of carcinogenesis and/or CRC progression. Towards this end, phages such as the ones we identified here could further support the growth of pathobionts, which can cause damage to intestinal cells, local inflammation and more. Thus, phages are increasingly acknowledged as a putative risk factor for CRC, whose importance has been underestimated in the past [31]. As to further mechanisms, phages might change homeostasis of the bacterial microbiome, targeting bacterial species or commensals, that are not pathogenic or oncogenic *per se*, favouring the expansion of bacteria that promote inflammation or even carcinogenesis [30]. Again, several studies indicate that phages are capable of inducing a ‘leaky gut’ [124], areas of increased intestinal permeability that enable the infiltration of pathogenic bacteria, further promoting chronic inflammation and possibly contributing to a spread of some species to immediate metastatic target organs that generally are considered to be sterile (see below) [124]. Finally, it has been shown that phage‐induced bacteriolysis causes the release of cellular debris into the microenvironment, inducing inflammation, whereby bacterial DNA and lipopolysaccharides are able to act as a pathogen‐associated molecular pattern (PAMP) that triggers immune response [30]. Phages, therefore, could represent an immunogenic stimulus in their own right and be associated with the release of PAMPs that further stimulate the immune system. Specifically, PAMPs will reach the liver first, inducing inflammation [125, 126]. The phage‐mediated bacteriolysis might induce a wave of endotoxins able to ignite the immune response, albeit its extent is not completely understood. Nevertheless, it has been hypothesized that such stimulation might be involved in establishing chronic inflammation [127, 128, 129, 130]. Tetz and Tetz [124] are the principal advocates of the possible pathogenic consequences of phage activity, and given the preliminary state of research on phage interaction with human physiology, it is not surprising that there is little literature besides the one cited of these two authors. Moreover, most of the descriptions of phage immunity have been rather inferred than clinically demonstrated. Still, it is logical to assume that phage‐mediated bacteriolysis will release endotoxins (including bacterial DNA, LPS, and peptidoglycan) in the intestinal lumen that are capable of activating immune cells of, for example of the intestinal wall. In contrast, intact whole bacteria, being adapted to their environment within their host, might minimize their immunogenicity to cause a low as possible activation of the immune system. Thus, we believe it to be more likely that the consumption of whole bacteria due to phage‐mediated bacteriolysis will rather increase than reduce the number of immunogens, and inflammation, in the intestinal milieu, although it is certainly acknowledged that other theories might be true as well, this maybe also depending on the type of phage and corresponding type of bacterial host.

Taken together, there are several mechanisms by which phages, and phage‐host interactions, impact the microbiome and pro‐inflammatory, or even pro‐carcinogenic/‐metastatic, conditions in the intestine. Thus, our findings can encourage mechanistic studies on particular bacteriophages as to their putative function in CRC carcinogenesis or progression in the future, especially since, despite the intense research ongoing to describe the microbial communities in the human intestinal tract of CRC and IBD [22, 24, 36, 38, 131], the characterization of the microbial ecosystem itself is still poorly understood.

Similar to the bacteria, many of the viral species we observed enriched in primary colorectal tumours and liver metastases, besides phages, have been reported to possess oncogenic and/or pro‐inflammatory potential. This is certainly true for EBV which we found in whole‐genome sequencing, including even in one metastasis after Blast filtering, and also by PCR in an independent series in which about a quarter of the analysed primary tumour/normal colorectal tissue sample pairs showed a specific positivity in the carcinomas. Regarding putative pro‐carcinogenic mechanisms, EBV has been reported to act several fold [132, 133]: it produces oncogenic proteins and micro‐interfering RNAs (miRNAs) which could cause pro‐oncogenic pathway switches within a human cell. Moreover, EBV can induce ‘hit‐and‐run mutagenesis’, thereby increasing frequencies of genetic mutations in the infected cell, hyper‐methylate tumour suppressor genes and disrupt cellular miRNA expression [132, 133]. In particular, EBV activates the Wnt/β‐catenin signalling pathway which is fundamental in CRC carcinogenesis and progression [134, 135]. Certainly, our own data are descriptive only and do not give experimental or functional evidence for EBV causing or promoting CRC, and in general, it is known that EBV infects up to 90% of the population [136]. Still, it is estimated that about 10% of all gastric carcinomas are causally associated with EBV infection [137]. In CRC primary tumour tissues, EBV has been recovered by others with a prevalence of between 5% and 60%, depending on the sensitivity of the method used [138, 139, 140], thus in ranges we have found in our sets. This is not surprising as the gut has been found to be an important reservoir of infected resting B cells [141]. These cells usually do not proliferate, but can be periodically reactivated in that they start producing virus, or initiate a short burst of cell growth during which viral products with oncogenic properties are generated [142]. Based on our data, we still consider it unlikely that, in most instances, EBV directly infects CRC cells and contributes to transformation through endogenous expression of viral oncogenes or of viral noncoding RNAs. However, EBV‐infected cells are known to secrete microvesicles that contain many viral products including LMP1, the main EBV oncogene, as well as noncoding RNAs such as the EBERs [143, 144]. Thus, it is theoretically possible that microvesicles laden with EBV‐derived molecules are captured by the neighbouring colonic epithelial cells and contribute to the acquisition of the malignant phenotype. Such a scenario remains entirely speculative but is worth investigating experimentally. Regarding further (microenvironmental) interactions of EBV, interestingly, it has been reported that EBV increases the infectivity of torque teno (TT) virus, a feature that might contribute to multiple sclerosis [145]. In our analysis, we retrieved torque teno virus sequences in a few CRC liver metastases and, even confirmed after stringent Blast filtering, primary CRCs, and although biases such as the known high blood volume in the liver could have impacted on these observations, a potential long‐term cooperation between these two viruses in the CRC context might be an interesting speculation, which needs to be investigated in future functional studies, especially since both torque teno virus and EBV can be present in the blood without causing obvious clinical symptoms of infection [146, 147, 148, 149, 150]. Indeed, TT virus is commonly encountered in both blood and faecal samples, and it is reported to be more prevalent in CRC and chronic inflammation than in normal tissues [151, 152].

We observed other viruses with oncogenic potential. CMV infects preferentially neoplastic epithelium, where it can reach a prevalence of over 40% compared to < 6% in the surrounding normal tissues [153], and tumour tissues have a much higher risk of being infected with CMV than normal tissues (OR = 6.6) [154]. CMV infection activates Wnt signalling pathways, promoting cell proliferation and migration [155]. HHV‐6B infects over 90% of the human population, can cause gastroenteritis, and it is detected in about 6% of colon carcinomas and 4% of rectal adenocarcinomas [156]. HHV‐6B infects mainly T lymphocytes (but also macrophages, dendritic cells, fibroblasts and epithelia) and has been associated with lymphoproliferative diseases as well as oral and cervical carcinomas [157]. HHV‐6 has a prevalence of about 90% [158, 159], but its association with IBD is controversial. For example, no significant difference has been reported for the prevalence of this virus between IBD patients and healthy controls [160]. Further studies also indicated a nonsignificant difference in HHV‐6 prevalence between IBD and controls, but, interestingly, co‐infection with EBV was significantly higher in IBD cases [161]. Other studies reported a prevalence of 4–44% in IBD [162, 163] and in five out of eight (62.5%) patients with colonic adenomas [164] but in none of healthy matched controls. HPyV7 has been isolated about one decade ago from the sera of healthy volunteers [165] and not extensively associated with human diseases yet; still, one study reported its presence in 54–62% of 37 thymic tumours, with 17 samples associated with a high expression of the large T antigen, as compared to no evidence for this virus in 20 fetal thymic tissues [166]. Although this comparison, certainly, might be biased by the use of nonmatched controls, it provides a first hint for a possible oncogenic role of HPyV7, potentially mediated by the large T antigen. Our observation of this virus in CRC in our present work certainly needs to be extended, and validated, by epidemiological and functional studies in the future [167]. Polyomavirus large T (which increases the stability of β‐catenin and, consequently, the activation of the Wnt signal pathway) and small t antigens (which can affect the expression of several modulators of the cell cycle including cyclin D, c‐myc, and survivin) [168], mediate the oncogenic ability of these viruses. Another polyomavirus, SV40, has been associated directly with colorectal cancer. A survey of 94 colon cancers identified the presence of SV40 in 6% of them while it was absent in the colon tissues of healthy controls, but infection was not statistically significant as a risk factor (OR = 3.91, *P*‐value = 0.115) [169].

HERV‐K113 is an endogenous retrovirus with a prevalence of up to 30% [170], and, unlike many other viruses of this class, it is still capable of reactivation and production of infectious virions [171]. Increased prevalence of HERV‐K113 has been reported in breast cancer tissues (16.7%) in comparison to matched normal tissues (12.7%) [172] and in multiple sclerosis (11.9%) compared to 4.6% in healthy controls, suggesting a role in cancer as well as in autoimmunity [173]. Reactivation of a related virus (HERV‐K‐T47D), measured by the expression of its reverse transcriptase, has been suggested as a biomarker for breast cancer [174]. Given the potential reactivation of this virus and its connection to cancer, it would have been interesting to measure the expression of HERV‐K113 at the protein level but, unfortunately, we had no tissue material left to perform this additional analysis. It is also possible to foresee HERV‐K113 and further infection‐associated markers being included in examining circulating tumour cells (CTC). These cells are gaining popularity as CRC diagnostic indicators [175]. Potentially, a study on the methylation status of HERV‐K113 genes or HERV‐K113 mRNAs within CTCs might help with cancer precursor diagnosis. Likewise, identifying genetic material belonging to oncoviruses either in the bloodstream or inside CTCs might help to stratify patients with CRC. Nevertheless, extensive clinical research is needed to sustain such possibilities.

We also observed EMCV, a zoonotic pathogen that has a seroprevalence ranging from two per cent to over 30% [176, 177]. EMCV is not known to cause cancer but it has been shown that it can induce autoimmune reactions against T cells, fostering the development of type 1 diabetes [178]. It can also interfere with the cellular E3 ubiquitin ligase E6‐associated protein (E6AP) [179], which regulates cell proliferation via the PI3K‐AKT signalling pathway [180]. In the present study, the detection of EMCV sequences in liver metastasis again raises the question whether this virus might be contributing to aspects of inflammation, carcinogenesis or microenvironmental interactions in CRC.

Taking advantage of the observed presence of microorganisms within our samples, we tried to apply statistical methods to distinguish between normal colorectal, primary colorectal carcinoma and metastasis tissue types. Previous studies have applied the Shannon index for diagnostic purposes, for instance applying the genetic variability of *c‐MYC* to identify breast cancer patients at a higher risk of mortality [181, 182, 183]. In our attempt, both viral and bacterial species proved to be useful in differentiating primary colorectal carcinoma from normal paired colorectal tissues, which supports the notion that our microbial findings in CRC primary tumours were not due to mere contamination by contents of the intestinal lumen or normal tissue fractions, but that microbial or metagenomic findings might aid in the differential diagnosis of malignant versus normal colorectal tissues.

The much lower Shannon indices observed in metastatic sections was unsurprising since these tissues are expected to be rather sterile. Thus, the presence of some microorganisms in liver metastases still appears counter‐intuitive. Only HERV‐K113, an endogenous retrovirus, displayed a profile characterized by high sequence coverage associated with integration in the host’s chromosomes (Fig. S1), thus, the other microorganisms must have gained access to the liver cells by means other than vertical transmission. The species we observed in metastases were not reported as environmental contaminants [184, 185]. Thus, the most likely explanation, as already indicated above, is systemic access through conditions like a ‘leaky gut’ or ‘focal leak’ (see above) and/or general access via the blood stream (see above). Indeed, an increasing number of studies is reporting the presence of, for example, phages in tissues previously considered sterile [30, 186, 187, 188]. We could not compare our results in metastases with previously existing metagenome/microbiome studies in these tissues since, to the best of our knowledge, our study has pioneering character in this regard so far. Therefore, future studies at also larger metastasis sample sizes are mandatory.

Certainly, our study has limitations, including methodological ones. First, it was initially designed for whole‐genome screening. Consequently, no viral enrichment, or general enrichment for microbial genomes, had been carried out, nor included, in the processing of the samples. Thus, the low microbial biomass could have introduced a bias in the results, generating lower than expected findings in sequence copies, or in individual sequence coverage, of microbial sequences as compared to sequences from human chromosomes [185]. Lower sensitivity might also have led to a lower than expected detection of some species, for example JCV or HPV, or a lower detection of positivity as compared to other methods such as PCR, although, still, our results on EBV correspond to the range of frequencies reported by others [138, 139, 189, 190, 191]. Second, except for EBV we had no further tissue available to perform further wet laboratory validations. Nonetheless, the fact that the species we observed have not been reported as contaminants, and that some of the species have been observed by others with similar frequencies, still strengthen our findings. Also, the parallelism between the presence of some bacterial hosts and phages in the same specimens support the validity of our results, since it is highly likely that host and guest species are present simultaneously in the same tissue. Such parallelism would not be expected if the sequences were mapped randomly or derived from environmental contaminants.

## 5 Conclusion

In conclusion, this is the first work carrying out metagenomic analysis on CRC metastases as compared to corresponding primary CRC tumours and normal colorectal tissues and that attempted to apply microbial richness calculations to differentiate these tissues. We showed that the microbial landscape might be used to differentiate primary colorectal tumours from nonmalignant tissue and metastases, in particular by using the Shannon index. Moreover, we highlighted particular species, especially including the previously not extensively considered species of (bacterio‐)phages, for future functional studies as to how they could contribute to colorectal carcinogenesis or even progression and metastasis, for example by creating permissive or nonpermissive microbial environments. Extending this analysis to broader sets of available samples, for example in multicentre approaches, could foster a better understanding of infectious agents as a potentially complex interplay of cofactors for CRC carcinogenesis and progression, and assess whether the use of microbial analysis could support precision medicine to pinpoint patients at increased risk for CRC or CRC metastasis.

## Conflict of interest

The authors declare no conflict of interest.

## Author contributions

LM mainly performed and coordinated the analyses within this paper. LM, TR, and JJML performed all aspects of the bioinformatics analysis, MLA acquired the samples and consents, coordinated the aspects associated with their collection, and led wet laboratory sample analysis and PCR. SD and HJD performed and interpreted EBER stainings. HA initiated the idea of virome, phage and bacteriome analysis in association with CRC carcinogenesis and metastasis, and coordinated the research and funding. All authors participated in the discussion and interpretation of data, the writing and/or revision, and approval of the manuscript.

## Supporting information


**Fig. S1**. Visualization of viral integration of human endogenous retrovirus K113 (HERV‐K113).
**Table S1**. Read counts stratified by tissue type.

## Data Availability

Anonymized sequence data are stored within the EGA database (accession number EGAS00001002717) as already described previously [41].
