# Supplementary material for: Metagenomic analysis of primary colorectal carcinomas and their metastases identifies potential microbial risk factors
Source: Mol Oncol. 2021 Aug 30;15(12):3363–84. doi: 10.1002/1878-0261.13070 (PMC8637581; doi:10.1002/1878-0261.13070)
Supplement: Supplementary file 1 — Fig. S1. Visualization of viral integration of human endogenous retrovirus K113 (HERV‐K113). Table S1. Read counts stratified by tissue type. [file MOL2-15-3363-s001.pdf]

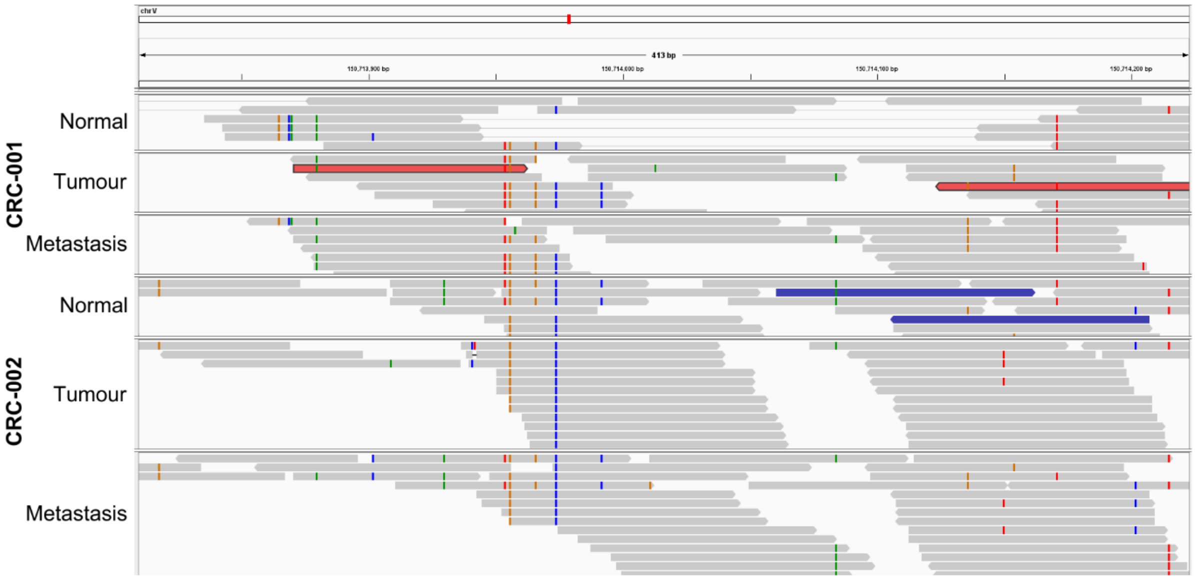

Suppl. Fig. 1. Visualization of viral integration of human endogenous retrovirus K113 (HERV-K113). Example of HERV-K113 mapping to the viral genome observed in two patients (CRC001 and CRC-002), each with a trio of normal colon, primary colorectal tumour, and liver metastases tissues. The upper part of the figure shows the artificial viral chromosome (ChrV) obtained by concatenating the genomes of 10,384 viruses, with the vertical red bar indicating the relative position within ChrV of the window of 143 nucleotides represented in the lower part of the figure. The lower part shows the alignment of the sequences (horizontal red bars) obtained by whole-genome sequencing analysis. The number of overlapping sequences represents the coverage of a given position. If a virus is integrated into the human chromosomes, it will be present at the same rate as the human genomes (average coverage here: 28.33), which is the case for HERV-K113, suggesting the integration of this virus. The coloured vertical bars in the lower part of the figure represent point mutations.

Suppl. Tab. 1. Read counts stratified by tissue (normal colon, primary colorectal tumour, and metastases, all except one case* liver metastases), coverage, map on the combined human and viral genome (including the properly paired fraction), map on the viral genomes only (raw), and filtered by blast alignment.

ID	Tissue	Coverage	Mapped	Paired	Raw	Filtered
CRC-001	Normal	21.01	779,397,638	778,605,033	300,798	41
	Tumour	23.72	896,697,974	876,094,251	253,369	54
	Metastasis	21.68	805,144,453	804,468,293	277,357	105
CRC-002	Normal	26.01	936,079,958	935,287,433	351,351	135
	Tumour	29.94	1,079,271,673	1,078,764,616	369,370	109
	Metastasis	27.98	1,008,685,786	1,008,251,678	412,905	77
CRC-003	Normal	27.13	982,716,164	981,971,563	341,459	133
	Tumour	26.39	966,694,949	952,586,003	392,586	153
	Metastasis	24.46	879,207,189	878,812,190	421,911	106
CRC-004	Normal	21.88	809,961,359	808,590,329	169,237	37
	Tumour	23.50	859,107,264	858,698,577	278,476	48
	Metastasis*	23.93	871,171,587	870,765,537	273,478	66
CRC-005	Normal	24.07	900,077,840	894,313,916	310,689	96
	Tumour	24.40	911,369,182	910,189,223	274,055	77
	Metastasis	22.57	840,015,001	839,617,534	247,069	65
CRC-006	Normal	28.03	1,018,199,477	1,017,726,620	366,773	118
	Tumour	28.66	1,040,473,204	1,039,983,717	316,752	93
	Metastasis	28.69	1,045,172,871	1,044,705,882	317,503	84
CRC-007	Normal	110.30	3,911,696,560	3,885,312,466	933,138	579
	Tumour	0.06	2,088,708	2,079,005	3,244	0
	Metastasis	54.8	1,938,146,693	1,931,803,009	794,909	243
CRC-008	Normal	30.07	1,475,461,382	1,473,293,013	3,608	43
	Tumour	6.8	258,767,127	258,225,606	2,458	19
	Metastasis	40.94	1,650,547,553	1,648,181,520	2,449	40
CRC-009	Normal	27.13	969,377,475	968,812,774	453,638	96
	Tumour	26.39	1,033,819,939	1,033,354,717	293,122	45
	Metastasis	24.46	924,277,632	923,852,284	283,739	41
CRC-010	Normal	28.60	1,035,732,054	1,035,261,070	385,441	126
	Tumour	28.4	1,025,719,304	1,025,271,172	379,141	82
	Metastasis	25.7	940,518,663	940,132,382	302,218	51
CRC-011	Normal	29.92	1,089,082,375	1,087,181,322	316,749	133
	Tumour	28.17	1,019,788,712	1,019,192,980	337,252	80
	Metastasis	26.20	945,330,703	944,159,934	339,072	77
CRC-012	Normal	25.69	935,507,282	934,969,254	321,960	64
	Tumour	26.30	953,170,594	952,164,051	337,122	91
	Metastasis	26.04	943,092,032	942,638,079	354,361	78
		28.33 ± 16.21 (Mean)	*37,681,568,357*	*37,585,317,033* (Total)	*11,518,759*	*3,485*

* Lung tissue.
